# Side effects of hormone therapy in patients with malignant breast neoplasm: a cross-sectional study

**DOI:** 10.1590/0034-7167-2024-0536

**Published:** 2025-12-08

**Authors:** Erilaine de Freitas Corpes, Romel Jonathan Velasco Yanez, Cristina Poliana Rolim Saraiva dos Santos, Monalisa Dutra Barbosa, Mayara Maria Silva da Cruz Alencar, Paulo César de Almeida, Ana Fátima Carvalho Fernandes, Régia Christina Moura Barbosa Castro

**Affiliations:** I Universidade Federal do Ceará. Fortaleza, Ceará, Brazil.; II Universidade Estadual do Ceará. Fortaleza, Ceará, Brazil

**Keywords:** Breast Neoplasms, Tamoxifen, Aromatase Inhibitors, Drug-Related Side Effects and Adverse Reactions, Cross-Sectional Studies, Neoplasias de la Mama, Tamoxifeno, Inhibidores de la Aromatasa, Efectos Colaterales y Reacciones Adversas Relacionados con Medicamentos, Estudios Transversales

## Abstract

**Objectives:**

to evaluate the side effects of hormone therapy in patients with malignant breast neoplasia.

**Methods:**

a cross-sectional, analytical study conducted in a mastology outpatient clinic in Ceará, Brazil. Seventy-five women with breast cancer using tamoxifen or aromatase inhibitors were included and classified into four subgroups based on the type and duration of therapy. Fisher's exact test was used to assess the association between associated factors and the occurrence of side effects.

**Results:**

women using tamoxifen for >12 months had more sexual complaints. Hot flashes, musculoskeletal pain, and fatigue predominated in the anastrozole groups. There was a statistically significant association between side effects and daily lifestyle habits, previous treatment, and calcium replacement therapy.

**Conclusions:**

all groups presented some side effects and there are some factors that can intensify these symptoms, therefore, health professionals should monitor these effects to intervene early.

## RESUMEN

**Objetivos:**

evaluar los efectos secundarios de la terapia hormonal en pacientes con cáncer de mama.

**Métodos:**

estudio transversal en un ambulatorio de mastología en Ceará, Brasil, con 75 mujeres que usaban tamoxifeno o inhibidores de la aromatasa, divididas según tipo y duração del tratamiento. Se utilizó la prueba exacta de Fisher para analizar asociaciones.

**Resultados:**

el tamoxifeno por más de 12 meses se asoció con mayores quejas sexuales. En los grupos con anastrozol, fueron comunes los sofocos, dolor musculoesquelético y fatiga. Se observó relação significativa entre los efectos secundarios y factores como hábitos diarios, tratamientos previos y suplementação de calcio.

**Conclusiones:**

todos los tratamientos hormonales evaluados presentaron efectos secundarios. Factores individuales pueden intensificar estos síntomas, lo que destaca la necesidad de que los profesionales de salud realicen un seguimiento constante para intervenir tempranamente y mejorar la calidad de vida de las pacientes.

## INTRODUCTION

In 2020, the number of new breast cancer cases among the global female population reached 2,261,419 (24.5%), ranking fourth worldwide and being the most common cancer among women^(1)^. In Brazil, it is the most frequently diagnosed cancer in all regions, with an estimated 73,610 new cases in 2023, corresponding to an estimated risk of 66.54 new cases per 100,000 women^(2)^.

In the context of oncological treatment, Hormone Therapy (HT) stands out as a recommended therapeutic approach for women with malignant breast neoplasms that test positive for estrogen receptors (ER) or progesterone receptors (PR). Among the types of HT, tamoxifen (TMX) is highlighted as a selective ER modulator recommended for premenopausal women^(3)^, while aromatase inhibitors (AIs), such as exemestane, letrozole, and anastrozole (ANTZ), are used for postmenopausal women with breast cancer. The main action of AIs is the inhibition of aromatase, which prevents estrogen production^(4)^.

Approximately three-quarters of breast cancers are positive for hormone receptors, which is directly associated with improved disease-free survival and reduced mortality rates^(5)^. Among its advantages, HT does not require hospitalization for administration, reducing hospitalization costs, equipment use, and human resources; it also minimizes interference in the patient's social life and increases their autonomy over the therapy^(6)^.

However, this therapy also has numerous side effects, which are the primary reason for reduced adherence or nonadherence to treatment, increasing the chances of recurrence and disease-related mortality. A systematic review conducted in 2021 analyzed survivors' experiences with breast cancer and the side effects of HT. The most commonly reported side effects included fatigue, hot flashes, night sweats, musculoskeletal pain, weight gain, alopecia, and sexual dysfunctions, such as loss of sexual interest, vaginal dryness, and dyspareunia^(7)^.

Thus, this research is justified by the need to understand the influence of the side effects caused by HT on the lives of women with breast neoplasms, as these factors can directly impact treatment, reduce adherence, and worsen the disease prognosis. Additionally, this study is relevant as it aligns with the third Sustainable Development Goal (SDG), which aims to ensure healthy lives and promote well-being for all at all ages^(8)^.

## OBJECTIVES

To evaluate the side effects resulting from hormone therapy in patients with malignant breast neoplasms.

## METHODS

### Ethical Aspects

The project was submitted to the Research Ethics Committee of the Assis Chateaubriand Maternity School, part of the Federal University of Ceará, and received approval.

### Study design, Period and Location

This article is derived from a dissertation with a cross-sectional, analytical design and a quantitative approach, developed following the criteria established in the Strengthening the Reporting of Observational Studies in Epidemiology (STROBE) guidelines^(9)^.

The study was conducted with patients receiving care at the Mastology Outpatient Clinic of the Assis Chateaubriand Maternity School, a reference service for women with breast neoplasms. Data collection took place between September and December 2022.

### Population, inclusion, and exclusion criteria

The study included women aged 18 years or older, diagnosed with malignant breast neoplasms, and undergoing HT with TMX or AI.

Exclusion criteria were: women undergoing chemotherapy and/or radiotherapy concomitantly with HT, as other therapeutic modalities for cancer could influence HRQoL; women with a prior or current history of other cancers; the presence of metastases, which is a factor contributing to reduced HRQoL^(10)^; and women with cognitive impairment, confirmed by a medical diagnosis, which would prevent understanding of the instruments used.

The sample size was calculated using the formula for finite populations, based on a prior study conducted at the same institution that compiled data on patients undergoing HT between March 2020 and July 2021. This earlier study identified a total of 114 women with luminal A or B breast cancer^(11)^. A confidence level of 95% (standard deviation of 1.96) and a maximum margin of error of 5% were used. For "p" and "q", a percentage of 50% was assumed to account for a more heterogeneous sample. Applying the formula for finite populations resulted in a desired sample size of 88 women. However, the final sample for this study consisted of 75 patients due to challenges encountered during data collection, as described in the study's limitations.

The patients were divided into four subgroups based on the type and duration of HT use, as follows: 20 patients in the ANTZ > 12 months subgroup; 20 patients in the ANTZ â‰¤ 12 months subgroup; 20 patients in the TMX > 12 months subgroup; and 15 patients in the TMX â‰¤ 12 months subgroup. This time frame was chosen as it is commonly used in studies evaluating HRQoL and HT^(12)^.

### Study protocol

Initially, a form encompassing sociodemographic and clinical data was administered^(11)^. Regarding side effects, those identified in Peddie's study were listed and assessed dichotomously^(7)^.

The lead researcher conducted weekly reviews of patient records at the mastology outpatient clinic to identify eligible participants. Subsequently, these patients were approached on the day of their respective appointments, during which recruitment occurred according to the inclusion and exclusion criteria. After consenting to participate and signing the informed consent form (ICF), the interview was conducted.

Each interview lasted approximately 10 minutes. It is worth noting that the data collection instruments were administered by the lead researcher and two trained assistant researchers, who had received prior training on the study and the application of the instruments.

### Analysis of results and statistics

The collected data were processed using SPSS 20.0, license number 10101131007. For numerical variables, the mean, median, and standard deviation were calculated. Absolute and relative frequencies were calculated for categorical variables. Fisher's exact test was used to assess the association between related factors and the occurrence of side effects, with a significance level of 5% established for all inferential analyses.

## RESULTS

Between September and December 2022, 1,520 patients were seen at the mastology outpatient clinic. Of these, 110 were undergoing HT, and 75 comprised the sample for this study. The reasons 35 patients were not included in the sample were: refusal to participate in the study; inability to complete the form; discontinuation of treatment; and absence from the consultation.

### Demographic and clinical characteristics related to the disease

The mean age was 53.6 years (Â±10.5), ranging from 32 to 78 years. Most participants were from rural areas of Ceará (53.3%), had a partner (57.3%), and had up to two children (57.3%). Regarding clinical characteristics, invasive ductal carcinoma (IDC) accounted for 85.3% of the sample. Stage II (48%) was the most prevalent, with 25.3% corresponding to stage IIA and 22.7% to stage IIB. The most common luminal subtype was luminal B (48%), followed by luminal A (34.7%) and the hybrid luminal subtype (17.3%) (Table 1).

**Table 1:** Distribution of women according to demographic and clinical characteristics, Fortaleza, Ceará, Brazil, 2023.

Characteristics	n	%	MeanÂ±SD
Age Range (years)			53.6 Â± 10.5
32 : 49	28	37.3	
50 : 59	27	36.0	
60 : 78	20	26.7	
Marital status			
With partner	43	57.3	
Without partner	32	42.7	
Number of children			4 Â± 1.2
None	8	10.7
1 to 2	43	57.3	
3 or more	24	32.0	
Histology			
Invasive ductal carcinoma	64	85.3	
Ductal carcinoma in Situ	4	5.3	
Invasive lobular carcinoma	3	4.0	
Others	4	5.3	
Staging			
IA	16	22.9	
IIA	19	27.1	
IIB	17	24.3	
IIIA	13	18.6	
IIIB	3	4.3	
0	2	2.9	
Luminal			
Luminal A	26	34.7	
Luminal B	36	48.0	
Luminal hybrid	13	17.3	

When assessing comorbidities, 42.7% had one or more types of comorbidities, the most prevalent being Systemic Arterial Hypertension (30.7%), followed by Diabetes Mellitus (24%) and Dyslipidemia (13.3%). Regarding social habits, 13.3% reported social alcohol consumption and a previous and/or current history of smoking. Of the participants, 52% reported not having an active sex life.

### Side effects of Hormone Therapy

**Figure 1:**
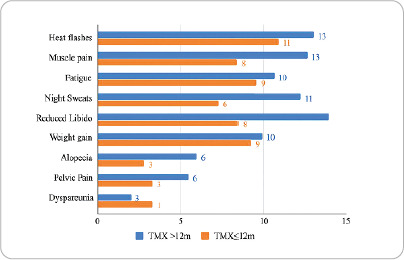
Distribution of side effects caused by Tamoxifen and duration of use, Fortaleza, Ceará, Brazil, 2023.

**Figure 2:**
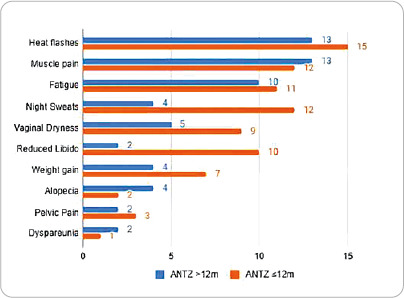
Distribution of side effects caused by Anastrozole and time of use, Fortaleza, Ceará, Brazil, 2023.

Women who took TMX had more sexual complaints, especially those who used TMX >12m. In the ANTZ group, hot flashes, musculoskeletal pain and fatigue predominated, and the ANTZ â‰¤12m subgroup had more side effects than the ANTZ >12m subgroup (Figures 1 and 2).

.

.

There was an association (p<0.05) between side effects and daily lifestyle habits, previous HT treatment and calcium replacement. Alcoholic women had more night sweats than non-alcoholic women (p<0.047), weight gain and musculoskeletal pain were greater among women undergoing chemotherapy (p<0.037/ p<0.005), night sweats and musculoskeletal pain were more present in women undergoing calcium replacement (p<0.005/ p<0.003) (Table 2).

**Table 2:** Factors associated with the occurrence of side effects, Fortaleza, Ceará, Brazil, 2023.

Side effects	Factors associated														
	Alcoholism					Chemotherapy					Calcium replacement				
	Absent		Present			Absent		Present			Absent		Present		
	N	%	N	%	*p*	N	%	N	%	*p*	N	%	N	%	*p*
Night sweats	2	20.0	8	80.0	0.047*	27	49.1	28	50.9	0.795	23	69.7	10	30.3	0.005*
Weight gain	6	60.0	4	40.0	1.000	29	52.7	26	47.3	0.037*	45	40.0	30	60.0	0.158
Pain Musculoskeletal	6	60.0	4	40.0	0.147	23	41.8	32	58.2	0.005**	10	30.3	23	69.7	0.003**

* Fisher's Exact Test;.

** Fisher-Freeman-Halton Test.

## DISCUSSION

The side effects of HT are the primary reason for reduced adherence or non-adherence to treatment, which increases the likelihood of recurrence and mortality from the disease. In this study, hot flashes and night sweats were prevalent in both groups, particularly among those taking ANTZ. In addition to the physiological changes caused by treatment, which result from hypothalamic thermoregulatory imbalance that alters serotonin and norepinephrine levels^(13)^, it is believed that climatic conditions may also intensify these symptoms, given that the patients in this study live in a tropical country.

Hot flashes and night sweats are commonly reported by women using TMX^(14)^, but those on ANTZ also experienced these symptoms. In an RCT of women taking ANTZ and exemestane, hot flashes and night sweats were among the five most common side effects, particularly during the first three months of therapy^(15)^.

Musculoskeletal pain and fatigue were also predominant in the ANTZ groups. Similar findings were observed in the BIG 1-98 study, where these side effects were more frequent with ANTZ therapy compared to TMX^(16)^. Comparable data were also found in other studies^(17,18)^.

In the context of musculoskeletal pain and fatigue, it is important to highlight that the hormonal changes induced by HT lead to bone mass loss, resulting in joint pain, fatigue, and an increased risk of fragility fractures, which negatively impact HRQoL by potentially impairing individuals' ability to perform daily activities.

As a result of these side effects, assessing calcium levels is essential to improve patients' quality of life. In this study, 42% of women did not take calcium supplements, which may have contributed to the occurrence of musculoskeletal pain. In a study conducted in Turkey, patients with lower levels of 25-hydroxy vitamin D had increased joint pain intensity^(18)^.

Vitamin D can be used in cancer patients and may positively influence HRQoL, increase survival, and reduce treatment-related side effects. However, supplementation should be monitored by a physician, and routine tests should be conducted to assess the need for vitamin D and calcium supplementation^(19,20)^.

Regarding sexual complaints, vaginal dryness was more commonly reported in the TMX >12m and ANTZ â‰¤12m subgroups. A multicenter study conducted in English-speaking health centers identified that women undergoing adjuvant endocrine therapy experienced sexual problems for up to two years, with vaginal dryness being the most frequently reported symptom^(21)^. In the ATAC study, a precursor to research evaluating the side effects of HT for breast neoplasms, the ANTZ group reported more vaginal dryness, dyspareunia, and reduced libido compared to the TMX group^(22)^.

Reduced sexual interest was observed equally in the TMX >12m and ANTZ â‰¤12m subgroups, while dyspareunia was predominant in the TMX group, regardless of duration of use. These side effects are commonly reported by women using endocrine therapy for breast cancer and can affect self-esteem, lead to distancing from partners, and directly impact relationships^(23,24)^. In a study conducted in Sweden, women reported not only experiencing these symptoms but also feeling unable to share them with friends or family out of fear of not being understood^(24)^.

Although several studies have demonstrated that these side effects are recurrent in women using hormonal therapy for breast cancer, some factors may contribute to the exacerbation of these symptoms. In this study, the side effects were analyzed in conjunction with several factors believed to influence symptom occurrence, including comorbidities, alcoholism, smoking, chemotherapy, radiotherapy, surgery, and calcium supplementation.

After data analysis, associations were found between night sweats and alcoholism (p<0.047), night sweats and calcium supplementation (p<0.005), musculoskeletal pain and calcium supplementation (p<0.003), musculoskeletal pain and chemotherapy (p<0.005), and weight gain and chemotherapy (p<0.037).

Women who underwent chemotherapy experienced greater weight gain compared to those who did not undergo this treatment. Weight gain during chemotherapy is frequently reported among patients and has a multifactorial cause, as chemotherapeutic agents alter body composition by causing muscle depletion, contributing to fluid retention in cells^(25)^, potentially increasing hunger and appetite, and inducing menopause, which reduces metabolism and contributes to weight gain.

This finding suggests that prior treatments should be considered when initiating HT. Such weight gain can negatively affect HRQoL, as it is not only associated with excess body fat but also with increased symptoms such as fatigue, muscle pain, the development of noncommunicable chronic diseases, and reduced self-esteem.

Women who underwent chemotherapy also reported higher levels of musculoskeletal pain. Several factors may influence the incidence of bone pain, such as the chemotherapy regimen, the malignancy stage, prior therapies, and the patient's bone condition before treatment. Women exposed to taxanes tend to experience greater musculoskeletal and neuropathic pain^(26)^.

Regarding musculoskeletal pain, an association was observed between this variable and calcium supplementation, indicating that women taking calcium supplements reported more musculoskeletal pain. Conversely, a study conducted in India showed that supplementation with vitamin D3 and calcium reduced hormone therapy-induced arthralgia^(27)^. This highlights the need to supplement not only calcium but also vitamin D, which helps the body effectively absorb calcium. Moreover, pain assessment is subjective, as various factors may influence its intensity. In this case, the patient's bone condition prior to treatment could exacerbate pain.

Although statistically significant, no studies were found that confirm the relationship between reduced night sweats and calcium supplementation or increased night sweats and alcohol consumption in women treated for breast neoplasms. It is known that alcohol acts as a vasodilator, increasing bodily fluid production and contributing to sweating^(28)^. However, no studies confirm the relationship between these two variables and breast cancer.

This underscores the need to manage these side effects, as there are factors that can be modified or controlled to reduce their exacerbation, such as musculoskeletal pain. Strategies such as dietary changes, daily physical activity, wearing lightweight clothing to alleviate sweating symptoms, and taking breaks during household chores to reduce fatigue are recommended^(29)^.

### Study limitations

A limitation of this study was the small sample size, which was reduced due to challenges encountered at the study site and during the data collection period, including strikes at the healthcare institution and the World Cup. These events led to the rescheduling of appointments and, consequently, the loss of patients for the study sample. However, a plan was developed to recruit these patients and include as many women as possible undergoing hormonal therapy at the institution where the study was conducted.

### Contributions to the healthcare field

Measuring and monitoring the side effects caused by HT in the context of breast cancer enable the provision of more comprehensive and higher-quality care and may reduce the harm caused by therapy. Therefore, this study reinforces the necessity and importance of sequentially monitoring these women throughout their HT treatment.

## CONCLUSIONS

The side effects of hot flashes, musculoskeletal pain, night sweats, and sexual complaints were the most frequently reported by patients. Although these symptoms were present in all four subgroups, they were more common in the TMX >12m and ANTZ â‰¤12m subgroups. Alcohol consumption and calcium supplementation influenced the occurrence of night sweats and musculoskeletal pain, while chemotherapy influenced weight gain and musculoskeletal pain.

## Data Availability

The research data are available within the article.
