# Supplementary material for: Side effects of hormone therapy in patients with malignant breast neoplasm: a cross-sectional study
Source: Rev Bras Enferm. 2025 Dec 8;78(4):e20240536. doi: 10.1590/0034-7167-2024-0536 (PMC12716806; doi:10.1590/0034-7167-2024-0536)
Supplement: Translation

# Translation: Efeitos colaterais da hormonioterapia em pacientes com neoplasia maligna mamária: estudo transversal

**DOI:** 10.1590/0034-7167-2024-0536pt

**Authors:** Erilaine de Freitas Corpes, Romel Jonathan Velasco Yanez, Cristina Poliana Rolim Saraiva dos Santos, Monalisa Dutra Barbosa, Mayara Maria Silva da Cruz Alencar, Paulo César de Almeida, Ana Fátima Carvalho Fernandes, Régia Christina Moura Barbosa Castro

**Affiliations:** I Universidade Federal do Ceará. Fortaleza, Ceará, Brazil.; II Universidade Estadual do Ceará. Fortaleza, Ceará, Brazil

**Keywords:** Neoplasias da Mama, Tamoxifeno, Inibidores da Aromatase, Efeitos Colaterais e Reações Adversas Relacionados a Medicamentos, Estudos Transversais

## Abstract

**Objetivos:**

avaliar os efeitos colaterais decorrentes da hormonioterapia em pacientes com neoplasia maligna da mama.

**Métodos:**

estudo transversal, analítico realizado em ambulatório de mastologia no Ceará, Brasil. Foram incluídas 75 mulheres com câncer de mama em uso de tamoxifeno ou inibidor da aromatase, sendo classificadas em quatro subgrupos com base no tipo e na duração da terapia. Foi utilizado o teste exato de Fisher para avaliar a associação entre os fatores associados e a ocorrência de efeitos colaterais.

**Resultados:**

as mulheres em uso de tamoxifeno >12m apresentaram mais queixas sexuais. Nos grupos anastrozol predominaram os fogachos, dores musculoesqueléticas e fadiga. Houve associação estatisticamente significante entre efeitos colaterais e hábitos de vida diários, tratamento anterior e reposiação de cálcio.

**Conclusões:**

todos os grupos apresentaram algum efeito colateral e há alguns fatores que podem intensificar esses sintomas, portanto, os profissionais de saúde devem monitorar esses efeitos para intervir precocemente.

## INTRODUÇÃO

Em 2020, o número de casos novos de câncer de mama na população feminina mundial correspondeu a 2.261.419 (24,5%), ocupando o quarto lugar no ranking mundial e sendo o tipo mais comum na população feminina^(1)^. No Brasil, é o diagnóstico de câncer mais frequente em todas as regiões, com estimativa de 73.610 casos novos em 2023, o que corresponde a um risco estimado de 66,54 casos novos por 100 mil mulheres^(2)^.

No contexto do tratamento oncológico, a hormonioterapia (HT) destaca-se como abordagem terapêutica recomendada para mulheres com neoplasias malignas de mama que apresentam resultado positivo para receptores de estrogênio (RE) ou progesterona (RP). Entre os tipos de HT, destaca-se o tamoxifeno (TMX), um modulador seletivo de RE recomendado para mulheres na pré-menopausa^(3)^, e os inibidores de aromatase (IAs), utilizados por mulheres na pós-menopausa com câncer de mama. Sua principal ação é a inibiação da IA, impedindo a produação de RE, sendo eles o exemestano, o letrozol e o anastrozol (ANTZ)^(4)^.

Cerca de três quartos dos cânceres de mama são positivos para receptores hormonais, o que está diretamente relacionado à melhora da sobrevida livre de doença e à reduação das taxas de mortalidade^(5)^. Dentre as vantagens da HT, destaca-se a não necessidade de internação para sua administração, reduzindo custos com hospitalização, equipamentos e recursos humanos; além disso, ela proporciona menor interferência na vida social da paciente e aumenta sua autonomia sobre a terapia^(6)^.

No entanto, essa terapia também apresenta muitos efeitos colaterais, sendo esta a principal razão para a reduação ou não adesão ao tratamento, o que aumenta as chances de recorrência e mortalidade pela doença. Um estudo de revisão sistemática desenvolvido em 2021 analisou na literatura as experiências de sobreviventes de câncer de mama sobre os efeitos colaterais da terapia hormonal, e os mais relatados foram: fadiga, ondas de calor, suores noturnos, dor musculoesquelética, ganho de peso, alopecia e disfunções sexuais, incluindo perda de interesse sexual, secura vaginal e dispareunia^(7)^.

Logo, esta pesquisa se justifica pela necessidade de compreender a influência que os efeitos colaterais ocasionados pela HT têm na vida das mulheres com neoplasia mamária, pois esses fatores podem afetar diretamente o tratamento, reduzir a adesão e piorar o prognóstico da doença. Além disso, este estudo é relevante porque está alinhado com o terceiro Objetivo de Desenvolvimento Sustentável, que se baseia em garantir uma vida saudável e promover o bem-estar para todos em todas as idades^(8)^.

## OBJETIVOS

Avaliar os efeitos colaterais decorrentes da hormonioterapia em pacientes com neoplasia maligna da mama.

## MÉTODOS

### Aspectos Éticos

O projeto foi encaminhado ao Comitê de Ética em Pesquisa (CEP) da Maternidade Escola Assis Chateaubriand, pertencente à Universidade Federal do Ceará, e obteve aprovação.

### Desenho, período e local do estudo

Este artigo é oriundo de uma dissertação, com desenho do tipo transversal e analítico, de abordagem quantitativa, que utilizou, para a elaboração do relatório de pesquisa, os critérios estabelecidos no *Strengthening the Reporting of Observational Studies in Epidemiology* (STROBE)^(9)^.

O estudo foi realizado com pacientes em acompanhamento no Ambulatório de Mastologia da Maternidade Escola Assis Chateaubriand, um serviço de referência no atendimento à s mulheres com neoplasia mamária. A coleta de dados ocorreu entre os meses de setembro e dezembro de 2022.

### População, critérios de inclusão e exclusão

Foram incluídas mulheres com idade igual ou superior a 18 anos, diagnosticadas com neoplasia maligna da mama, em uso de HT do tipo TMX ou IA.

Foram excluídas mulheres em tratamento quimioterápico e/ou radioterápico concomitante com a HT, pois a realização de outras modalidades terapêuticas para o câncer pode influenciar na Qualidade de Vida Relacionada à Saúde (QVRS); mulheres com história prévia ou atual de outros cânceres; presença de metástase, uma vez que este é um fator que contribui para a reduação da QVRS^(10)^; e mulheres que apresentassem comprometimento da capacidade cognitiva, comprovada por diagnóstico médico, que impossibilitasse a compreensão dos instrumentos.

A amostra foi calculada pela fórmula para populações finitas, tendo como base um estudo desenvolvido anteriormente na mesma instituiação, que forneceu os dados referentes à s pacientes em uso de HT no período de março de 2020 a julho de 2021. Esse estudo evidenciou um total de 114 mulheres com câncer de mama luminal A ou B^(11)^. Dessa forma, foi estabelecido nível de confiança de 95% (expresso em desvio-padrão como 1,96) e erro máximo de 5%. Para "p" e "q", utilizou-se o percentual de 50% para amostras mais heterogêneas. Após a aplicação da fórmula para populações finitas, chegou-se ao valor desejado de 88 mulheres. No entanto, a amostra final deste estudo foi composta por 75 pacientes, devido a percalços durante a coleta dos dados, descritos nas limitações deste estudo.

As pacientes foram divididas em quatro subgrupos, de acordo com o tipo e o tempo de uso da HT, resultando em: 20 pacientes no subgrupo ANTZ > 12 meses; 20 pacientes no subgrupo ANTZ â‰¤ 12 meses; 20 pacientes no subgrupo TMX > 12 meses; e 15 pacientes no subgrupo TMX â‰¤ 12 meses. A escolha desse período foi definida por ser um dos intervalos de tempo utilizados nos estudos que avaliam QVRS e o uso de HT^(12)^.

Dois aspectos importantes devem ser ressaltados: primeiramente, no grupo IA, todas as mulheres faziam uso exclusivamente de ANTZ; e, em segundo lugar, as pacientes do grupo TMX â‰¤ 12 meses foram coletadas em sua totalidade, perfazendo um total de 15 pacientes.

### Protocolo do estudo

Inicialmente, foi aplicado um formulário que englobava dados sociodemográficos e clínicos^(11)^. Quanto aos efeitos colaterais, foram listados os encontrados no estudo de Peddie e respondidos de forma dicotômica^(7)^.

Primeiramente, a pesquisadora principal checava semanalmente os prontuários das pacientes agendadas no ambulatório de mastologia para identificar aquelas elegíveis para o estudo. Posteriormente, essas pacientes foram abordadas no dia de suas respectivas consultas, ocasião em que ocorreu o recrutamento de acordo com os critérios de inclusão e exclusão. Após o aceite de participação e assinatura do Termo de Consentimento Livre e Esclarecido (TCLE), realizou-se a entrevista.

Cada entrevista teve duração média de 10 minutos. Vale ressaltar que os instrumentos de coleta de dados foram aplicados pela pesquisadora principal e por duas pesquisadoras auxiliares, que receberam treinamento prévio sobre o estudo e a aplicação dos instrumentos.

### Análise dos resultados e estatística

Os dados coletados foram processados usando o SPSS 20.0, número de licença 10101131007. Para as variáveis numéricas, foram calculadas a média, a mediana e o desvio padrão. As frequências absolutas e relativas foram calculadas para as variáveis categóricas. Foi utilizado o teste exato de Fisher para avaliar a associação entre os fatores associados e a ocorrência de efeitos colaterais, estabelecendo-se um nível de significância de 5% para todas as análises inferências.

## RESULTADOS

No período de setembro a dezembro de 2022, foram atendidas 1520 pacientes no ambulatório de mastologia, das quais 110 faziam uso de HT e, destas, 75 compuseram a amostra deste estudo. Os motivos pelos quais 35 pacientes não foram incluídas na amostra foram: recusa em participar da pesquisa; falta de condições para responder ao formulário; descontinuidade do tratamento; e ausência na consulta.

### Características demográficas e clínicas relacionadas à doença

A média de idade foi de 53,6 anos (Â±10,5), variando entre 32 e 78 anos. A maioria das participantes era procedente do interior do Ceará (53,3%), tinha companheiro (57,3%) e até dois filhos (57,3%). Com relação à s características clínicas, o carcinoma ductal invasivo (CDI) representou 85,3% da amostra. O estágio II (48%) foi o mais prevalente, com 25,3% correspondendo ao estágio IIA e 22,7% ao estágio IIB. O subtipo luminal mais comum foi o luminal B (48%), seguido pelo luminal A (34,7%) e pelo híbrido luminal (17,3%) (Tabela 1).

**Tabela 1:** Distribuiação das mulheres conforme as características demográficas e clínicas, Fortaleza, Ceará, Brasil, 2023.

Características	n	%	MédiaÂ±DP
Faixa etária (ano)			53,6 Â± 10,5
32 : 49	28	37,3	
50 : 59	27	36,0	
60 : 78	20	26,7	
Estado civil			
Com companheiro	43	57,3	
Sem companheiro	32	42,7	
Número de filhos			4 Â± 1,2
Nenhum	8	10,7	
1 a 2	43	57,3	
3 ou +	24	32,0	
Histologia			
Carcinoma Ducal Invasivo	64	85,3	
Carcinoma Ductal In Situ	4	5,3	
Carcinoma Lobular Invasivo	3	4,0	
Outros	4	5,3	
Estadiamento			
IA	16	22,9	
IIA	19	27.1	
IIB	17	24.3	
IIIA	13	18.6	
IIIB	3	4.3	
0	2	2.9	
Luminal			
Luminal A	26	34,7	
Luminal B	36	48,0	
Luminal híbrido	13	17,3	

Ao avaliar as comorbidades, 42,7% das participantes apresentaram um ou mais tipos de comorbidade, sendo a mais prevalente a hipertensão arterial sistêmica (30,7%), seguida por diabetes mellitus (24%) e dislipidemia (13,3%). Com relação aos hábitos sociais, 13,3% relataram consumo social de álcool e histórico anterior e/ou atual de tabagismo. Além disso, 52% das participantes relataram não ter uma vida sexual ativa.

### Efeitos colaterais da HT

As mulheres que usaram TMX apresentaram mais queixas sexuais, especialmente aquelas que utilizaram TMX por mais de 12 meses. No grupo ANTZ, predominaram as ondas de calor, dor musculoesquelética e fadiga, sendo que o subgrupo ANTZ â‰¤12 meses apresentou mais efeitos colaterais do que o subgrupo ANTZ >12 meses (Figuras 1 e 2).

**Figura 1:** Distribuiação dos efeitos colaterais ocasionados pelo Tamoxifeno e tempo de uso, Fortaleza, Ceará, Brasil, 2023.

.

**Figura 2:** Distribuiação dos efeitos colaterais ocasionados pelo Anastrozol e tempo de uso, Fortaleza, Ceará, Brasil, 2023.

.

Houve uma associação significativa (p<0,05) entre os efeitos colaterais e os hábitos de vida diários, tratamentos anteriores à HT e reposiação de cálcio. As mulheres que consumiam álcool apresentaram mais suores noturnos do que aquelas que não consumiam (p<0,047). O ganho de peso e a dor musculoesquelética foram mais frequentes entre as mulheres submetidas à quimioterapia (p<0,037 e p<0,005, respectivamente). Além disso, suores noturnos e dor musculoesquelética foram mais prevalentes nas mulheres que realizaram reposiação de cálcio (p<0,005 e p<0,003, respectivamente) (Tabela 2).

**Tabela 2:** Fatores associados com a ocorrência de efeitos colaterais, Fortaleza, Ceará, Brasil, 2023.

Efeitos Colaterais	Fatores Associados														
	Etilismo					Quimioterapia					Reposiação de Cálcio				
	Ausente		Presente			Ausente		Presente			Ausente		Presente		
	N	%	N	%	*p*	N	%	N	%	*p*	N	%	N	%	*p*
Sudorese Noturna	2	20,0	8	80,0	0,047*	27	49,1	28	50,9	0,795	23	69,7	10	30,3	0,005*
Ganho de Peso	6	60,0	4	40,0	1,000	29	52,7	26	47,3	0,037*	45	40,0	30	60,0	0,158
Dor Musculoesquelética	6	60,0	4	40,0	0,147	23	41,8	32	58,2	0,005**	10	30,3	23	69,7	0,003**

* Teste Exato de Fisher;.

** Teste Fisher-Freeman-Halton.

## DISCUSSÃO

Os efeitos colaterais da HT são a principal razão para a reduação ou não adesão ao tratamento, aumentando as chances de recorrência e mortalidade pela doença. Neste estudo, as ondas de calor e os suores noturnos foram prevalentes em ambos os grupos, mas especialmente entre aquelas que utilizaram ANTZ. Além das alterações fisiológicas causadas pelo tratamento, decorrentes do desequilíbrio termorregulatório hipotalâmico que afeta os níveis de serotonina e norepinefrina^(13)^, acredita-se que a situação climática também possa intensificar a ocorrência desses sintomas, considerando que as pacientes deste estudo vivem em um país tropical.

As ondas de calor e a sudorese noturna são comumente relatadas por mulheres em uso de TMX^(14)^, mas aquelas em uso de ANTZ também vivenciaram esses sintomas. Em um Ensaio Clínico Randomizado (ECR) realizado com mulheres que utilizavam ANTZ e exemestano, ondas de calor e suores noturnos estavam entre os cinco efeitos colaterais mais comuns, especialmente nos primeiros três meses de terapia^(15)^.

A dor musculoesquelética e a fadiga também foram predominantes nos grupos ANTZ. Dados semelhantes foram encontrados no estudo BIG 1-98, que demonstrou maior incidência desses efeitos colaterais na terapia com ANTZ em comparação ao TMX^(16)^. Resultados similares também foram observados em outros estudos^(17,18)^.

No contexto da dor musculoesquelética e da fadiga, vale ressaltar que a alteração hormonal que ocorre em decorrência da HT induz à perda de massa óssea, causando dor nas articulações, cansaço e aumento do risco de fraturas por fragilidade, com impactos negativos na QVRS, pois pode deixar o indivíduo incapaz de realizar suas atividades de vida diária.

Como resultado desses efeitos colaterais, a avaliação dos níveis de cálcio no corpo é essencial para proporcionar uma melhor QVRS ao paciente. Nesse estudo, 42% das mulheres não faziam reposiação de cálcio, podendo este ser um fator de contribuiação para a ocorrência de dor musculoesquelética. No estudo realizado na Turquia, os pacientes com níveis mais baixos de vitamina D, 25-hidroxivitamina D, tiveram um aumento na intensidade da dor articular^(18)^.

A vitamina D pode ser usada em pacientes com câncer e pode influenciar positivamente a QVRS, aumentar a sobrevida e reduzir os efeitos colaterais causados pelo tratamento. No entanto, a suplementação deve ser monitorada por um médico, e exames de rotina devem ser realizados para avaliar a necessidade de uso de vitamina D e cálcio^(19,20)^.

Em termos de queixas sexuais, a secura vaginal foi mais comumente relatada nos subgrupos TMX >12m e ANTZ â‰¤12m. Um estudo realizado em vários centros de saúde, tendo o inglês como idioma prioritário, identificou que as mulheres em uso de terapia endócrina adjuvante tiveram problemas sexuais por até dois anos de tratamento, sendo a secura vaginal o principal sintoma relatado^(21)^. No estudo ATAC, precursor das pesquisas que avaliam os efeitos colaterais da HT para neoplasia mamária, o grupo do ANTZ apresentou mais secura vaginal, dispareunia e diminuiação da libido do que o grupo do TMX^(22)^.

A reduação do interesse sexual foi a mesma nos subgrupos TMX >12m e ANTZ â‰¤12m, mas a dispareunia foi predominante no grupo TMX, independentemente do tempo de uso. Esses efeitos colaterais são comumente relatados por mulheres que usam TE para câncer de mama e podem afetar a autoestima, além de causar distanciamento do parceiro, afetando diretamente o relacionamento^(23,24)^. Em um estudo realizado na Suécia, as mulheres relataram que, além de sentirem esses sintomas, não conseguiam compartilhar esses efeitos colaterais com amigos ou familiares por medo de não serem compreendidas^(24)^.

Embora vários estudos tenham demonstrado que esses efeitos colaterais são recorrentes em mulheres que usam terapia hormonal para câncer de mama, alguns fatores podem contribuir para a exacerbação desses sintomas. Nesse estudo, os efeitos colaterais foram cruzados com vários fatores que se acredita influenciarem a ocorrência desses sintomas. São eles: comorbidade, alcoolismo, tabagismo, quimioterapia, radioterapia, cirurgia e reposiação de cálcio.

Após a análise dos dados, foi encontrada uma associação entre suor noturno/etilismo (p<0,047), suor noturno/reposiação de cálcio (p<0,005), dor musculoesquelética/reposiação de cálcio (p<0,003), dor musculoesquelética/quimioterapia (p<0,005) e ganho de peso/quimioterapia (p<0,037).

As mulheres que foram submetidas à quimioterapia apresentaram maior ganho de peso em comparação com aquelas que não realizaram esse tratamento. O ganho de peso durante a quimioterapia é frequentemente relatado entre os pacientes e tem causa multifatorial, uma vez que os quimioterápicos alteram a composiação corporal, causando depleação muscular, contribuem para a retenação de líquidos nas células^(25)^, podem aumentar a fome, despertando o desejo de comer, e induzem a menopausa, reduzindo o metabolismo e contribuindo para o ganho de peso.

Esse achado sugere que os tratamentos anteriores devem ser levados em consideração ao iniciar a HT. Esse ganho de peso pode afetar negativamente a QVRS, pois não está relacionado apenas ao excesso de gordura corporal, mas também ao aumento de sintomas como fadiga, dor muscular, desenvolvimento de doenças crônicas não transmissíveis e reduação da autoestima.

As mulheres que foram submetidas à quimioterapia tiveram mais dor musculoesquelética. Há vários fatores que podem influenciar a incidência de dor óssea, como o regime de quimioterapia, o estágio da malignidade, as terapias anteriores e a condiação óssea da paciente antes do tratamento. Mulheres expostas a taxanos tendem a apresentar mais dor musculoesquelética e neuropática^(26)^.

Ainda com relação à dor musculoesquelética, houve uma associação entre essa variável e a reposiação de cálcio, mostrando que as mulheres que faziam reposiação de cálcio tinham mais dor musculoesquelética. Por outro lado, um estudo realizado na Índia mostrou que a suplementação de vitamina D3 e cálcio reduziu a artralgia induzida pela terapia hormonal^(27)^. Isso destaca a necessidade de repor não apenas o cálcio, mas também a vitamina D, que ajuda o corpo a absorver o cálcio de forma eficaz. Além disso, a mensuração da dor é individual, pois há fatores que podem influenciar sua intensidade e, neste caso, a condiação óssea do paciente antes do tratamento pode influenciar a exacerbação da dor.

Apesar de estatisticamente significativos, não foram encontrados estudos que comprovassem a relação entre a reduação da sudorese noturna e a reposiação de cálcio, bem como o aumento da sudorese noturna e o consumo de álcool em mulheres que foram submetidas ao tratamento da neoplasia mamária. Sabe-se que o álcool atua como vasodilatador, aumentando a produação de fluidos corporais e contribuindo para a ocorrência de sudorese^(28)^. No entanto, não há estudos que comprovem a relação entre essas duas variáveis e o câncer de mama.

Isso reforça a necessidade de gerenciar esses efeitos colaterais, pois há fatores que podem ser modificados ou controlados para reduzir sua exacerbação, como a dor musculoesquelética. Dessa forma, são recomendadas estratégias como mudanças na dieta, atividade física diária, uso de roupas finas para reduzir os sintomas de sudorese e pausas durante as tarefas domésticas para minimizar a fadiga^(29)^.

### Limitações do estudo

Destaca-se como limitação deste estudo o tamanho da amostra, que foi reduzido devido aos desafios encontrados no local do estudo e durante o período de coleta de dados disponível, como greves na instituiação de saúde e a Copa do Mundo. Esses eventos resultaram na remarcação de consultas e, consequentemente, na perda de pacientes para compor a amostra do estudo. No entanto, foi desenvolvido um plano para recrutar essas pacientes e incluir o maior número possível de mulheres em uso de terapia hormonal na instituiação onde o estudo estava sendo realizado.

### Contribuições para a Área da Saúde

A mediação e o monitoramento dos efeitos colaterais causados pela HT no contexto do câncer de mama possibilitam a prestação de um atendimento abrangente e de maior qualidade e podem reduzir os danos causados pela terapia. Portanto, este estudo reforça a necessidade e a importância de monitorar essas mulheres sequencialmente durante todo o tratamento com HT.

## CONCLUSÕES

Os efeitos colaterais ondas de calor, dor musculoesquelética, suores noturnos e queixas sexuais foram os mais frequentemente relatados pelas pacientes. Embora esses sintomas estivessem presentes em todos os quatro subgrupos, eles foram mais frequentes nos subgrupos TMX >12m e ANTZ â‰¤12m. O alcoolismo e a reposiação de cálcio influenciaram a ocorrência de suores noturnos e dor musculoesquelética, enquanto a quimioterapia influenciou o ganho de peso e a dor musculoesquelética.

## Data Availability

Os dados da pesquisa estão disponíveis no corpo do artigo.
